# Notice of forthcoming erratum

**DOI:** 10.1100/tsw.2003.73

**Published:** 2003-08-22

## Abstract

The recently published peer-reviewed protocol article entitled Purification of Islets of Langerhans from Porcine Pancreas by John M. Graham (TheScientificWorldJOURNAL (2002) 2, 1657—1661, ISSN 1537-744X; DOI 10.1100/tsw.2002.847) is being revised. TheScientificWorldJOURNAL will shortly publish the article as an erratum entitled Iodixanol Density Gradient Preparation in University of Wisconsin Solution for Porcine Islet Purification. Links between the published paper and erratum will be made to facilitate cross-referencing.22 August 2003TheScientificWorldJOURNAL

